# Evaluating the Modified Patient Health Questionnaire-2 and Insomnia Severity Index-2 for Daily Digital Screening of Depression and Insomnia: Validation Study

**DOI:** 10.2196/45543

**Published:** 2023-05-22

**Authors:** Jae Won Oh, Sun Mi Kim, Deokjong Lee, Nak-Hoon Son, Jinsun Uh, Ju Hong Yoon, Yukyung Choi, San Lee

**Affiliations:** 1 Department of Psychiatry Yongin Severance Hospital, Yonsei University College of Medicine Yongin Republic of Korea; 2 Department of Nursing Andong Science College Andong Republic of Korea; 3 Department of Psychiatry and the Institute of Behavioral Science in Medicine Yonsei University College of Medicine Seoul Republic of Korea; 4 Department of Statistics Keimyung University Daegu Republic of Korea; 5 Mobigen Co, Ltd Seongnam Republic of Korea; 6 Korea Electronics Technology Institute Seongnam Republic of Korea; 7 Department of Intelligent Mechatronics Engineering Sejong University Seoul Republic of Korea

**Keywords:** Patient Health Questionnaire-2, PHQ-2, Insomnia Severity Index, ISI-2, depression, insomnia, mobile health, mobile phone

## Abstract

**Background:**

The Patient Health Questionnaire-2 (PHQ-2) and Insomnia Severity Index-2 (ISI-2) are screening assessments that reflect the past 2-week experience of depression and insomnia, respectively. Retrospective assessment has been associated with reduced accuracy owing to recall bias.

**Objective:**

This study aimed to increase the reliability of responses by validating the use of the PHQ-2 and ISI-2 for daily screening.

**Methods:**

A total of 167 outpatients from the psychiatric department at the Yongin Severance Hospital participated in this study, of which 63 (37.7%) were male and 104 (62.3%) were female with a mean age of 35.1 (SD 12.1) years. Participants used a mobile app (“Mental Protector”) for 4 weeks and rated their depressive and insomnia symptoms daily on the modified PHQ-2 and ISI-2 scales. The validation assessments were conducted in 2 blocks, each with a fortnight response from the participants. The modified version of the PHQ-2 was evaluated against the conventional scales of the Patient Health Questionnaire-9 and the Korean version of the Center for Epidemiologic Studies Depression Scale–Revised.

**Results:**

According to the sensitivity and specificity analyses, an average score of 3.29 on the modified PHQ-2 was considered valid for screening for depressive symptoms. Similarly, the ISI-2 was evaluated against the conventional scale, Insomnia Severity Index, and a mean score of 3.50 was determined to be a valid threshold for insomnia symptoms when rated daily.

**Conclusions:**

This study is one of the first to propose a daily digital screening measure for depression and insomnia delivered through a mobile app. The modified PHQ-2 and ISI-2 were strong candidates for daily screening of depression and insomnia, respectively.

## Introduction

### Background

Depression is the leading cause of mental illness in the general population and in primary care, instigating a substantial global health-related burden [1]. It is characterized by sadness, the loss of interest, tiredness, poor concentration, feelings of guilt, and disturbed sleep or appetite. In severe cases, depression can be associated with chronic diseases, high use of health care services, suicide, shortened life expectancy, and reduced quality of life [2].

Insomnia (a common comorbid condition with depression) is associated with difficulties in initiating or maintaining sleep, and its prevalence related to a reduction in physical and mental health [3]. Clinical and epidemiological studies have highlighted that sleep disturbances are closely linked to major depression [4]. In addition, studies have reported a comparable prevalence of insomnia among 41% of patients diagnosed with depression, with additional research suggesting a potential bidirectional link between the 2 disorders [5].

Various methods have been used to assess and screen for the symptoms of depression and insomnia. Screening assessment does not confirm diagnosis; however, depending on the results, they might be used to determine the risk of the respondent and refer accordingly [6]. Therefore, it is essential that such measures are reliable and valid to ensure that the generated outcomes are clinically accurate [7]. Brief self-report measures have been developed and used to screen for common mental disorders, depression, and insomnia. A self-report measure of depression, Patient Health Questionnaire-9 (PHQ-9), which matches the Diagnostic and Statistical Manual of Mental Disorders, Fourth Edition criteria of major depressive disorder, has been evaluated as the most reliable screening tool for depression through various systemic reviews and meta-analyses [6,8,9]. Likewise, the Insomnia Severity Index (ISI), a self-report measure of insomnia, has been recognized to effectively detect sleep problems [10,11].

However, based on the interest in fewer screening questions and the efficacy of such measures of depression and insomnia, further shortened versions of the PHQ-9 and ISI have been presented and validated in the literature. The Patient Health Questionnaire-2 (PHQ-2) uses the first 2 questions of the PHQ-9 concerning the frequency of depressed mood and anhedonia over the past 2 weeks [12]. This has demonstrated good sensitivity for detecting major depression [13,14]. Similarly, the Insomnia Severity Index-2 (ISI-2) is a 2-item version of the ISI that grades one’s satisfaction with current sleep patterns and assesses the interference with daily functioning because of sleep patterns. This has been proposed as a strong candidate for a brief scale measuring insomnia, with good psychometric properties [15].

Despite the reliable and valid quality of brief self-report measures for screening, depression and insomnia have often been underdetected and undertreated within primary care settings and the general community [16,17]. Various factors, including low prioritization, stigma, and barriers to assessment, have been suggested as reasons for underreporting [18]. With the increase in the availability and adoption of smartphones, enhanced methods of using new digital technologies to access and engage health care treatment have been established globally [19-21]. This has introduced assessment methods and intervention delivery, as apps can be designed for self-assessment and assist patients in assessing and monitoring their symptoms [22]. The use of smartphone apps also allows for ecological momentary assessment (EMA), which increases the accuracy of self-report responses by reducing recall bias and preventing aggregation by measuring the key variables in natural environments and in real time [23,24]. Existing systematic reviews have identified that EMA methods for collecting data are effective and reasonable for measuring momentary mood and stress owing to the ubiquity and easy use of smartphones [25].

### Objective

In accordance with the growth in the ability of EMA, this study aimed to address the existing barriers to assessment. As existing PHQ-2 and ISI-2 screening assessments are reflective of the experience from the previous 2 weeks, this study aimed to enhance the EMA by implementing the PHQ-2 and ISI-2 daily via a smartphone app. In addition, a psychometric assessment of the daily PHQ-2 and ISI-2 scales was conducted to evaluate the validity of smartphone-based daily screening measures. In particular, the assessment of PHQ-2 has been conducted twice daily, in the morning and afternoon, to control for the potential diurnal variations of depressive symptoms, which can fluctuate depending on the time [26].

## Methods

### Participants

Patients from the psychiatric outpatient clinic of the Yongin Severance Hospital in South Korea were recruited for this study. An offline advertisement was posted on bulletin boards near the psychiatry department. To be eligible, the participants must (1) be outpatients of psychiatric department (patients do not necessarily require a clinical diagnosis of depression or insomnia), (2) be aged ≥18 years, (3) provide consent for voluntary research participation, and (4) have an Android smartphone with access to Wi-Fi or 4G or 5G internet connectivity.

### Ethics Approval

This study was approved by the Institutional Review Board of the Yongin Severance Hospital (institutional review board number 9-2020-0160) and conducted in accordance with the Declaration of Helsinki. All participants provided written informed consent before participation.

### Materials

#### PHQ-9 Cutoff Score

The PHQ-9 is a 9-item questionnaire that assesses the severity of depression. The scores range from 0 to 27, with each question scored between 0 (not at all) and 3 (nearly every day). This scale is a reliable and valid measure for screening depression severity. A validation study of the Korean version of the PHQ-9, which reported a cutoff score of 10 for screening depression, demonstrated 81.8% sensitivity and 89.9% specificity [27]. Henceforth, in this study, the Korean version of the PHQ-9, with a cutoff score of 10, was used to assess the level of depression within the participants.

#### Modified PHQ-2

The PHQ-2 is a reduced version of the existing PHQ-9 scale, which evaluates the frequency of depressive symptoms and anhedonia over the past fortnight, with each item scored between 0 (not at all) and 3 (nearly every day). The use of the PHQ-2 for screening depression has been previously validated with a threshold PHQ-2 score of ≥3, reporting a sensitivity of 83% and a specificity of 92% in detecting major depression [12]. A recent validation of the PHQ-2 has reported a more conservative approach of considering 2 as the threshold value, which has been presented in various studies [13,28]. This study used the same PHQ-2 scale items, which were “How often have you been bothered by the following? 1) Little interest or pleasure in doing things; 2) Feeling down, depressed or hopeless,” and a modified response options for participants to rate their symptoms daily, with scores ranging from 0 to 4 (never, rarely, sometimes, often, and always). The number of PHQ-2 response options was modified to be in line with other daily scales and enhance participants’ usability, considering that this was a daily measure over a period of 28 days.

#### Center for Epidemiologic Studies Depression Scale–Revised

The Center for Epidemiologic Studies Depression Scale–Revised is a revised version of the original Center for Epidemiologic Studies Depression, which is used to screen for depressive disorders. It is a 20-item measure that adequately assesses the fundamental symptoms of depression as described in the Diagnostic and Statistical Manual of Mental Disorders, Fourth Edition [29]. In this study, the validated Korean version of the Center for Epidemiologic Studies Depression Scale–Revised (K-CESD-R) was implemented, with a threshold of 13 to identify the level of depression [30].

#### ISI Scores

The ISI is a 7-item self-report questionnaire that assesses the nature, severity, and impact of insomnia. The factors evaluated were sleep onset, sleep maintenance and early morning awakening problems, sleep dissatisfaction, interference of sleep difficulties with daytime functioning, noticeability of sleep problems by others, and distress caused by sleep difficulties. The items are scored on a 5-point Likert scale ranging from 0 (no problem) to 4 (very severe problem). The total score is interpreted in 4 different categories: absence of insomnia (0-7), subthreshold insomnia (8-14), moderate insomnia (15-21), and severe insomnia (22-28). Scores >15 were considered to indicate insomnia symptoms. Studies have reported adequate psychometric properties of the current scale [16].

#### ISI-2 Scores

The ISI-2 is a 2-item version of the ISI scale, and 1 item asks participants’ satisfaction with their current sleep pattern and another item represents the amount of interference with daily functioning because of their current sleep pattern. The total scores of the 2 items ranged from 0 to 8, and the scale has been validated with a cutoff of 6 points, which is considered appropriate to detect insomnia disorder with a sensitivity score of 84% and a specificity score of 76% [15]. This study implemented the ISI-2 scale items asking the participants to rate their symptoms daily, with scores ranging from 0 (not at all) to 4 (all the time).

#### Mental Protector Mobile App

**Figure 1:**
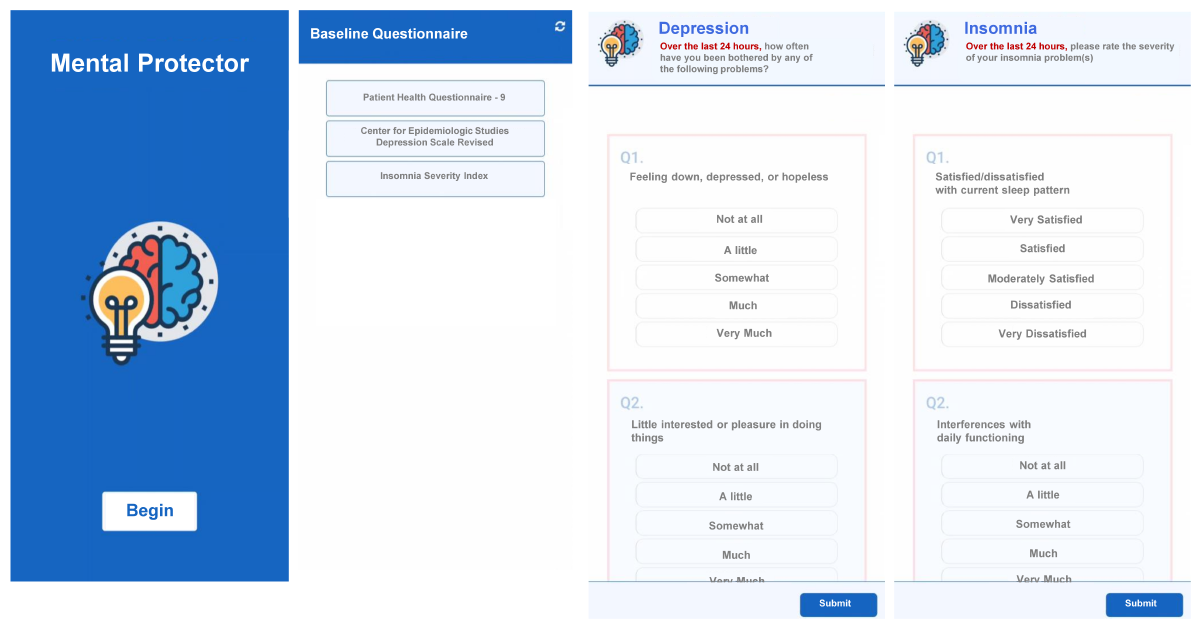
Screenshots of the Mental Protector app in the order of the main title page, baseline questionnaire page and pages for daily depression and insomnia rating, respectively.

The Mental Protector is a mobile app that comprises self-reporting depression- and insomnia-related scales designed to obtain data via an EMA [31]. Full scales of the K-CESD-R, PHQ-9, and ISI were administered at the baseline, midpoint (2 weeks after use), and end point (4 weeks after use). Modified PHQ-2 items were delivered daily to the participants through a push-alert method, once in the morning and once in the afternoon. Similarly, the ISI-2 scales were prompted to the participants, but only in the morning. The app was developed by a multidisciplinary team comprising a psychiatrist, researchers with expertise in eHealth delivery of interventions, and software programmers. The psychiatrist and researchers with experience in digital therapeutics provided advice on the study design and the protocol. On the basis of these guidelines, an IT firm named Mobigen Co developed the actual app for research. The initial language used was created in Korean, using the validated Korean version of the scales included. The screenshot image of the app used in this research has been translated into English and is presented in Figure 1.

### Procedures

**Figure 2:**
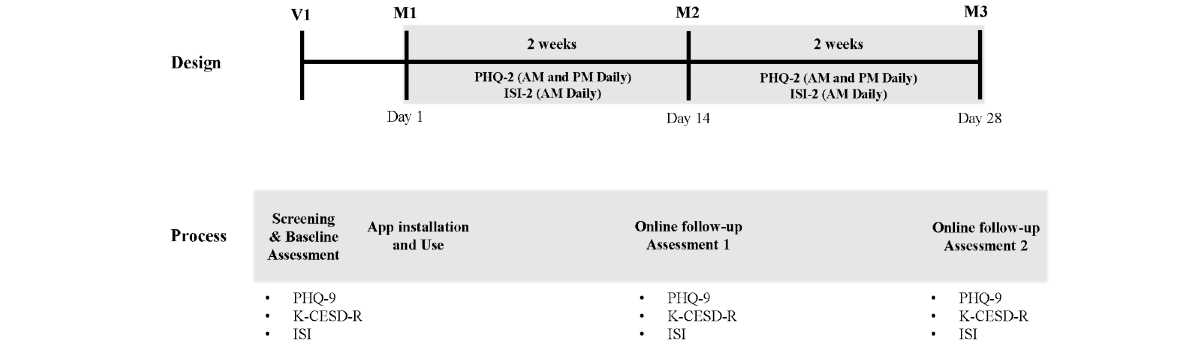
Study protocol outlining the design and process involved in this study. ISI: Insomnia Severity Index; K-CESD-R: Korean version of Center for Epidemiologic Studies Depression Scale–Revised; M1: mobile use time point 1; M2: mobile use time point 2; M3: mobile use time point 3; PHQ-9: Patient Health Questionnaire-9; V1: visit 1.

Participants were recruited from the Department of Psychiatry Outpatient Clinic at Yongin Severance Hospital. Eligible participants were introduced to the study by the research team, and informed consent was obtained. The participants were introduced to the mobile app (“Mental Protector”) that was installed on Android devices. In addition, participants’ basic sociodemographic information was obtained, and baseline assessments of the PHQ-9, K-CESD-R, and ISI were performed. These assessments were conducted under the supervision of the study’s researchers who provided appropriate instructions on site at the outpatient clinic. Once completed, the participants were explained about the daily prompt alerts delivered by the push function. A push alert for the modified PHQ-2 scale was sent twice daily: once in the morning and once in the late afternoon. Considering the differences in one’s depressive levels depending on the time of the day, the option to assess twice daily was decided [26]. Regarding ISI-2, the push alert was sent once in the morning, questioning the participants’ sleep satisfaction from the previous night. Furthermore, in cases where the participants did not respond to the daily screening items from the initial push alert, additional automated push alerts were sent up to 3 times in total to prompt participation. The study was conducted for 28 days from the day of the app installation. It has been designed in 2 blocks of 14-day assessments, as the conventional method of depression- and insomnia-screening assessments are performed with a reflection of the previous 14 days [32,33]. Hence, daily assessment is required over this period to determine the mean score for both the modified PHQ-2 and ISI-2. Daily assessments with 4 questions in the morning and 2 questions in the afternoon are estimated to take approximately 1 minute to complete, and follow-up assessments on days 14 and 28 are estimated to take 15 minutes. The details of the study design are presented in Figure 2.

### Statistical Analysis

The validation assessments were conducted in 2 blocks, each with a fortnightly response from the participants. On the basis of the PHQ-9 and K-CESD-R, the sensitivity and specificity of the modified PHQ-2 were calculated, and receiver operating characteristic (ROC) curve analysis was performed to identify the most appropriate threshold score for the modified PHQ-2. The mean score of the modified PHQ-2 was calculated from the assessment conducted in the morning and afternoon over a period of 2 weeks. Similarly, the sensitivity and specificity of ISI-2 were calculated based on the initial ISI response, and ROC curve analysis was used to identify the most appropriate threshold score for ISI-2. The mean score of the modified ISI-2 was calculated from the scores of the assessments conducted in the morning for a fortnight. Participants required 100% completion rates for the key biweekly measures—PHQ-9, K-CESD-R, and ISI—and >60% completion rates for daily assessments of the modified PHQ-2 and ISI-2 to be considered for analysis. SAS software (version 9.4; SAS Institute) was used for the analyses, and statistical significance was set at *P<*.05.

## Results

### Baseline Characteristics

A total of 201 outpatients participated in this study. Of the 201 outpatients, participant withdrawals and missing responses were removed, leaving 167 (83.1%) outpatients eligible for analysis in this study, 63 (37.7%) male patients and 104 (62.3%) female patients, aged between 18 and 69 years (mean 35.1, SD 12.1 years). This included the respondents with >60% of the daily assessment completion rates. There were no missing cases in the first 14 days of the daily assessment, and missing cases occurred during the 14- to 28-day period. On average, participants had 14.7 (SD 2.4) years of education. Regarding living arrangement status, 63.5% (106/167) of the patients were living alone and 36.5% (61/167) of the patients lived with cohabitants in their households. A total of 53.3% (89/167) of participants were currently employed, whereas 46.7% (78/167) were unemployed. Of the 167 participants, 86 (51.5%) participants had primary diagnosis of depression and 81 (48.1%) participants had other diagnoses, which included 33 (41%) patients with anxiety disorders, 19 (23%) patients with trauma and stress-related disorders, 10 (12%) patients with bipolar disorder, 6 (7%) patients with obsessive and compulsive disorder, 3 (4%) patients with psychosis, 8 (10%) patients with insomnia, 1 (1%) patient with attention-deficit/hyperactivity disorder, and 1 (1%) patient with bulimia nervosa. The basic sociodemographic details of the participants and the baseline measures of depression and insomnia are presented in Table 1, and the detailed breakdown of those with other than depression as primary diagnosis is presented in Multimedia Appendix 1.

**Table 1:** Demographic characteristics of participants (N=167).

Characteristics		Male participants (n=63)	Female participants (n=104)
**Primary diagnosis, n (%)**			
	Depression	30 (47.6)	56 (53.9)
	Other	33 (52.4)	48 (46.1)
**Age** **(years), n (%)**			
	18-25	33 (52.5)	19 (18.3)
	26-35	12 (19)	23 (22.1)
	36-45	12 (19)	32 (30.8)
	>45	6 (9.5)	30 (28.8)
**Education, n (%)**			
	High school and below	20 (31.7)	22 (21.1)
	University	26 (41.3)	63 (60.6)
	Graduate and above	17 (27)	19 (18.3)
**Current employment status, n (%)**			
	Unemployed	31 (49.2)	47 (45.2)
	Employed	32 (50.8)	57 (54.8)
**Socioeconomic status, n (%)**			
	High	4 (6.3)	5 (4.8)
	Mid	34 (54)	67 (65.1)
	Low	25 (39.7)	31 (30.1)
**Alcohol consumption, n (%)**			
	Nondrinker	37 (58.7)	67 (64.4)
	Drinker	26 (41.3)	37 (35.6)
**Smoking, n (%)**			
	Nonsmoker	38 (60.3)	71 (68.3)
	Past smoker	8 (12.7)	14 (13.4)
	Current smoker	16 (27)	19 (18.3)
**Living arrangement, n (%)**			
	Alone	50 (79.4)	56 (53.8)
	Together	13 (20.6)	48 (46.2)
**Baseline measures, mean (SD)**			
	PHQ-9^a^	12.4 (6.4)	12.2 (7.0)
	K-CESD-R^b^	30.6 (17.7)	32.8 (20.2)
	ISI^c^	12.6 (6.6)	13.4 (6.2)

^a^PHQ-9: Patient Health Questionnaire-9.

^b^K-CESD-R: Korean version of the Center for Epidemiologic Studies Depression Scale–Revised.

^c^ISI: Insomnia Severity Index.

### Reliability Analysis of Modified PHQ-2

Internal consistency assessment of the modified PHQ-2 scale was conducted at 2 time points: after week 2 and at the end of week 4. Correlational analyses were conducted with the PHQ-2 responses and the responses of the PHQ-9 and K-CESD-R assessed at the midpoint and end of week 2. This process was repeated to assess the internal consistency of the modified PHQ-2 morning and evening scores at weeks 3 and 4, with the PHQ-9 and K-CESD-R scores at the end of week 4. The correlation matrix with Pearson *r* values is presented in Table 2.

**Table 2:** Correlation matrix of depression with Patient Health Questionnaire-2 (PHQ-2).

				AM PHQ-2^a^		PM PHQ-2^b^		Mid–PHQ-9^c,d^		Mid–K-CESD-R^e,f^		End PHQ-9^g^		End K-CESD-R^h^
**Weeks 1-2 (*r* value)**														
	**AM PHQ-2**													
		*r*	1.00		0.95		0.79		0.78		—^i^		—	
		*P* value	—		<.001		<.001		<.001		—		—	
	**PM PHQ-** **2**													
		*r*	0.95		1.00		0.80		0.78		—		—	
		*P* value	<.001		—		<.001		<.001		—		—	
	**Mid–PHQ-9**													
		*r*	0.79		0.80		1.00		0.91		—		—	
		*P* value	<.001		<.001		—		<.001		—		—	
	**Mid–K-CESD-R**													
		*r*	0.78		0.78		0.91		1.00		—		—	
		*P* value	<.001		<.001		<.001		—		—		—	
**Weeks 3-4 (*r* value)**														
	**AM PHQ-2**													
		*r*	1.00		0.97		0.81		0.79		—		—	
		*P* value	—		<.001		<.001		<.001		—		—	
	**PM PHQ-2**													
		*r*	0.97		1.00		0.79		0.76		—		—	
		*P* value	<.001		—		<.001		<.001		—		—	
	**End PHQ-9^h^**													
		*r*	0.81		0.79		—		—		1.00		0.93	
		*P* value	<.001		<.001		—		—		—		<.001	
	**End K-CESD-R^i^**													
		*r*	0.79		0.76		—		—		0.93		1.00	
		*P* value	<.001		<.001		—		—		<.001		—	

^a^Morning PHQ-2 assessment.

^b^Afternoon PHQ-2.

^c^PHQ-9: Patient Health Questionnaire-9.

^d^PHQ-9 assessment at week 2.

^e^K-CESD-R: Korean version of the Center for Epidemiologic Studies Depression Scale–Revised.

^f^K-CESD-R assessment at week 2.

^g^PHQ-9 assessment at week 4.

^h^K-CESD-R assessment at week 4.

^i^Not applicable.

Cronbach α assessment of the modified PHQ-2 at week 2 presented a value of .94 and .94 for PHQ-9 and K-CESD-R, respectively, at midpoint (both *P*<.001) and .94 and .95 for PHQ-9 and K-CESD-R, respectively, at end point of week 4 (both *P*<.001), both identifying a high reliability for modified PHQ-2 measures in comparison with PHQ-9 and K-CESD-R. The tests showed a significant correlation between each depression scale score.

Test-retest reliability of the modified PHQ-2 scale was assessed by comparing the mean morning scores of the modified PHQ-2 between the first 2 weeks and the latter 2 weeks. This was assessed using the evening scores of the modified PHQ-2. The Pearson coefficient scores reported good test-retest reliability for reporting 0.89 and 0.90, respectively, with significant *P* values (both *P*<.001).

### Area Under the Curve and Cutoff Point of the Modified PHQ-2

**Figure 3:**
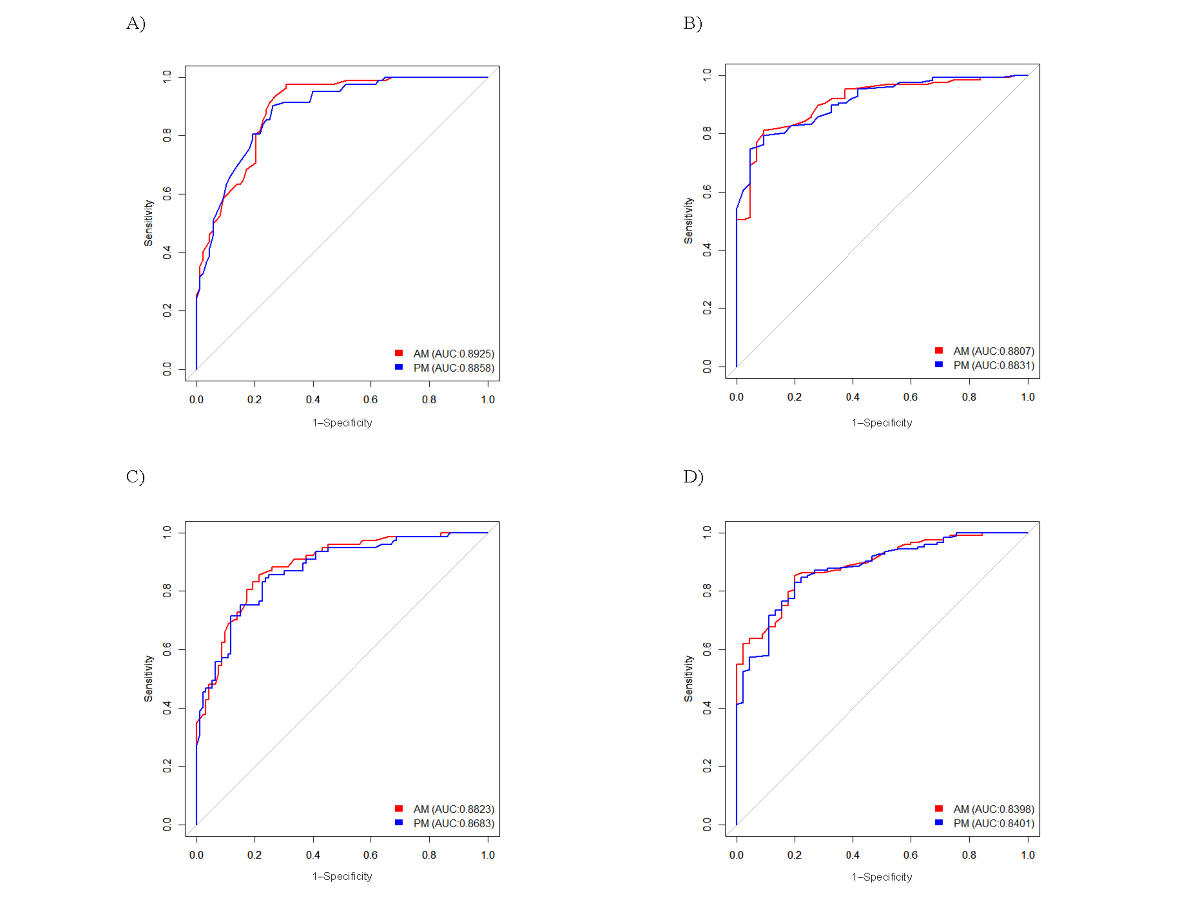
Receiver operating characteristics (ROC) curve for the modified Patient Health Questionnaire-2 (PHQ-2) daily rating scale, with the comparison of modified PHQ-2 morning (AM) and afternoon (PM) responses. They are displayed as follows: A) PHQ-9 at week 2; B) Korean version of Center for Epidemiologic Studies Depression Scale–Revised (K-CESD-R) at week 2; C) PHQ-9 at week 4; D) K-CESD-R at week 4. AUC: area under the curve.

The ROC curve evaluated the sensitivity and specificity of the modified PHQ-2 rating scale. The modified PHQ-2 daily rating scales were assessed twice with the PHQ-9 and twice with the K-CESD-R, each at 2 time points: once at the midpoint of week 2 and at the end of week 4. According to the area under the curve (AUC) values, there was no significant difference between the modified PHQ-2 screening conducted in the morning and afternoon. Thus, the ability to detect depression did not differ depending on the time of the PHQ-2 assessment. The details of the ROC assessment, including the AUC values, are shown in Figure 3.

The sensitivity and specificity values corresponding to the modified PHQ-2 cutoff scores are presented in Table 3. Here, a score of ≥10 for the PHQ-9 and 13 for the K-CESD-R detected depression. On the basis of ordering of cutoff values of all 8 assessments, including morning and afternoon at week 2 and week 4, and in comparison with the PHQ-9 and K-CESD-R scales, Youden J (YJ) statistics were used to determine the optimal threshold value. The YJ index summarizes the ROC curve by assessing the effectiveness of the diagnostic mark and enabling the selection of an optimal cutoff value [34]. Table 3 shows that 3.29 was most frequently identified cutoff value that had the highest YJ value and, therefore, was considered the acceptable cutoff value for daily screening of depression. Initially, 15 participants with low adherence rates, below 60%, were removed. The overall completion rate of the included 167 participants was 94.8%. Those with missing responses were from the second fortnight of the study; therefore, it is anticipated that the accuracy of the data from weeks 3 and 4 might have implications. Consequently, when establishing the optimal cutoff value, the preference for the results from the first 2 weeks was considered.

**Table 3:** Cutoff value of Patient Health Questionnaire-2 (PHQ-2) compared with Patient Health Questionnaire-9 (PHQ-9) and the Korean version of Center for Epidemiologic Studies Depression Scale–Revised (K-CESD-R).

Scale and cutoff value			Sensitivity	Specificity	YJ^a^
**Week 2**					
	**PHQ-2, AM^b^, compared with PHQ-9**				
		3.14	0.98	0.69	0.67
		3.29	0.94	0.73	0.67
		3.36	0.91	0.75	0.66
		3.21	0.96	0.69	0.65
		3.43	0.89	0.76	0.65
	**PHQ-2, PM^c^, compared with PHQ-9**				
		3.29	0.90	0.75	0.65
		3.21	0.91	0.71	0.63
		3.43	0.85	0.77	0.63
		3.50	0.84	0.79	0.63
		3.71	0.80	0.82	0.63
	**PHQ-2, AM, compared with K-CESD-R**				
		3.29	0.80	0.83	0.63
		3.14	0.83	0.79	0.62
		3.36	0.77	0.85	0.62
		3.21	0.82	0.79	0.61
		3.07	0.84	0.77	0.61
	**PHQ-2, PM** **, compared with** **K-CESD-R**				
		3.29	0.79	0.90	0.69
		3.36	0.75	0.90	0.65
		3.21	0.80	0.85	0.64
		3.43	0.74	0.90	0.64
		3.50	0.72	0.90	0.62
**Week 4**					
	**PHQ-2, AM** **, compared with PHQ-9**				
		3.67	0.86	0.78	0.63
		3.79	0.83	0.80	0.63
		3.91	0.80	0.82	0.62
		3.67	0.84	0.78	0.62
		3.78	0.83	0.79	0.62
	**PHQ-2, PM** **, compared with** **PHQ-9**				
		3.71	0.86	0.74	0.60
		3.78	0.84	0.76	0.60
		3.86	0.83	0.77	0.60
		4.08	0.75	0.84	0.59
		3.68	0.86	0.73	0.59
	**PHQ-2, AM** **, compared with** **K-CESD-R**				
		3.86	0.68	0.98	0.66
		3.79	0.70	0.96	0.66
		3.09	0.85	0.81	0.66
		3.00	0.87	0.79	0.66
		3.91	0.67	0.98	0.65
	**PHQ-2, PM** **, compared with** **K-CESD-R**				
		3.45	0.78	0.91	0.68
		3.41	0.80	0.89	0.68
		3.57	0.77	0.91	0.68
		3.43	0.79	0.89	0.67
		3.21	0.82	0.85	0.67

^a^YJ: Youden J statistic value.

^b^PHQ-2 assessed in the morning.

^c^PHQ-2 assessed in the afternoon.

### Reliability Assessment of ISI-2

In addition to the modified PHQ-2, an internal consistency assessment of the ISI-2 scale was also conducted, first after 2 weeks of use and then again at the end of week 4. Correlational analyses of the ISI-2 responses for the first 2 weeks were compared, with the ISI responses assessed at the midpoint and at the end of week 2. This process was repeated to assess the internal consistency of the ISI-2 at weeks 3 and 4 with the ISI scores at the end of week 4, the end point. Pearson *r* for the first 2 weeks was 0.78 and 0.81 for the last 2 weeks; Cronbach α was .88 and .89, respectively.

The test-retest reliability of the ISI-2 scale was also assessed by comparing the mean scores of the ISI-2 in the first 2 weeks with the latter 2 weeks. The Pearson coefficient scores reported good test-retest reliability, reporting 0.88 with a significant *P* value (*P*<.001).

### AUC and Cutoff Point of ISI-2

The ROC curve was used to evaluate the sensitivity and specificity of the daily ISI-2 rating scale. Each scale evaluation was performed twice, each at 2 time points, once at the midpoint of week 2 and the other at the end point of week 4. The AUC value was 0.85 and 0.88, respectively, for midpoint and end point assessment. The sensitivity and specificity values corresponding to various ISI-2 cutoff scores are presented in Multimedia Appendix 2. On the basis of assessments and use of the YJ index for the PHQ-2, a threshold value of 3.50 was considered acceptable to screen for insomnia when using the ISI-2. This cutoff value was consistent at the midpoint and end point, with the highest overall YJ score based on sensitivity and specificity analyses.

## Discussion

### Principal Findings

Traditional clinical assessments have relied heavily on retrospective questionnaires, asking respondents to recall their experiences and summarizing their responses to questions [35]. Previous research has shown that individuals with clinical depression show greater inaccuracy in their recall, which can have considerable clinical implications for the patient and their treatment experience [36]. Thus, this study proposed a method of digital daily screening to reduce any recall bias and observe the severity and frequency of conditions over time.

Using brief measures of depression and insomnia, namely, the modified PHQ-2 and ISI-2, respectively, this study presented a daily screening process to increase the accuracy of the EMA and reduce any recall bias. In addition, to justify that these digital daily screening scales are comparable with the existing measures, a psychometric validation of daily rating scales of the modified PHQ-2 and ISI-2 was conducted. Considering the ubiquitous nature of smartphone devices [37], these screening measures were delivered via smartphone apps. To control the possible variations in depressed mood depending on the time of day [26], screening assessments were conducted once daily in the morning and again in the afternoon for the depression-screening scale of the modified PHQ-2. Sleep quality was assessed once daily. The study was conducted with outpatient psychiatric patients, thus comprising a clinical population with depressive symptoms as their primary diagnosis or with other mental health conditions.

As per the study design, daily assessments were considered more accurate for screening of depressive symptoms than asking participants to reflect on the previous fortnight. From the psychometric evaluation of the modified PHQ-2 scale, the ROC curve findings reported no difference between the morning and afternoon responses of the modified PHQ-2 when assessed, in contrast to the traditional scales of the PHQ-9 and K-CESD-R. Unlike the anticipated diurnal variation, there was no significant disparity, suggesting that the use of the modified PHQ-2 was a viable measure for screening at any time of the day. Moreover, based on the reliability evaluations, the modified PHQ-2 scale showed good psychometric properties, demonstrating that it is a reliable screening measure for depression.

With the adaptation of the PHQ-2 as a daily screening measure, this study identified the mean cutoff score of the modified PHQ-2 when assessed for a period of 2 weeks. When assessed in comparison with both the PHQ-9 and K-CESD-R scales, for both morning and afternoon assessments of the modified PHQ-2, a mean value of 3.29 over a fortnight was considered appropriate to determine users with depressive symptoms. Existing literature has considered that PHQ-2 score of 2 or higher out of a total score of 6 to potentially screen for depression [13,28]. As this study validated a modified version and the modified scale had an additional response option, a cutoff value of 3.29 from a total score of 8 can be considered comparable with the scores derived from the initial validation studies.

The ISI, an insomnia-screening scale, has been developed into a shorter version of the ISI-2 in which users reflect on their sleep-related experiences from the previous 2 weeks. Similar to the PHQ-2 scale, this study proposed an alternative of rating the ISI-2 daily for a fortnight to screen for insomnia severity. This would assist in determining the severity and frequency of the symptoms across a 14-day period, providing a more precise clinical judgment for patients. Therefore, a psychometric assessment of the daily ISI-2 measures was conducted to evaluate its appropriateness for screening insomnia. Overall, the results demonstrated good internal consistency and test-retest reliability. When implementing ISI-2 as a daily screening measure for insomnia, an optimal cutoff score of 3.50 was presented according to sensitivity and specificity values. Unlike the PHQ-2 validation, the ISI-2 had a considerable difference in its cutoff value with the existing validation research. A previous study demonstrated that individuals with insomnia have sleep-related attention bias, which can influence the misperception of their sleep and the extent of their impairment in daytime activities [38]. Hence, owing to the risk of attention and recall bias, assessing insomnia and daytime impairment symptoms daily might create the possibility of a conservative approach, even with lower scores potentially indicating the risk of insomnia, unlike prior assessments that rely on a weekly or fortnightly recall.

### Strengths and Limitations

This study had important strengths. Before this study, there had been no consideration of using the PHQ-2 and ISI-2 to screen for depression and insomnia daily. This was the first study to propose the daily use of these scales with detailed psychometric evaluations. On the basis of this study’s findings, both the PHQ-2 and ISI-2 can be used daily with the use of smartphone technology to screen for depression and insomnia, implementing the mean cutoff values provided. This will benefit clinicians and researchers by improving the reliability of patient responses and reducing recall bias. In addition, the use of daily scoring of the PHQ-2 and ISI-2 will provide the benefit of being able to observe daily changes in users’ ratings of their depressive and insomnia symptoms using smartphone apps.

Regardless of the strengths identified, this study had some limitations. The validation process of the depression-screening scale, PHQ-2, involved the assessment and evaluation of 2 robust scales: PHQ-9 and K-CESD-R. Nonetheless, the PHQ-2 is a shortened version of the PHQ-9 and thus includes items that overlap with the scales. This means that an objective comparison was only performed using the K-CESD-R. Additional comparisons with other depression-screening scales could have enforced the validity of the new scale. Similarly, the ISI-2 is a shortened version of the original ISI. Including additional well-renowned scales such as the Pittsburgh Sleep Quality Index in the validation process would have strengthened the process. Furthermore, this study focused only on the recruitment of a clinical population. Further research in the general population may be necessary to validate these scales for general use. In addition, this study validated the daily rating scale by comparing daily measures with those of the conventional retrospective scale. Further use of daily assessments may provide opportunities for diverse validation studies on daily rating measures. Finally, although there were no missing cases in the first fortnight of the research period, the study included cases with missing responses between days 14 and 28. This would have influenced the validity and robustness of the process, which was addressed by identifying the cutoff values primarily from the data of the first fortnight. Nonetheless, there were no missing data during the first 14 days of the study, and the data collected during this period were considered more reliable and accurate. As a result, the preference for the results regarding the modified PHQ-2 cutoff values was determined from the first 2 weeks of assessment. Having no missing data in the first 14 days potentially demonstrates the importance of brief and easy user interface to retrain user participation.

### Conclusions

This study implemented the use of the modified PHQ-2 and ISI-2 and successfully validated these as daily digital screening scales, providing cutoff scores to be used to screen for depression and insomnia symptoms measured daily over a period of 14 days. This is in accordance with the existing assessment of depression and insomnia symptoms, which is conducted as a reflection of the previous fortnight [32,33]. Therefore, this study provides a new alternative for such screening measures and demonstrates the efficacy of using smartphone apps to obtain such momentary data. Medical practitioners may implement the proposed modified PHQ-2 and ISI-2 daily screening assessments for depression and insomnia to accurately assess the patients daily and use the mean values of both measures to present clinical findings that are equivalent to the conventional screening measures used to assess depressive and insomnia symptoms.

## Appendix

Multimedia Appendix 1 Participants with other diagnosis.

Multimedia Appendix 2 Cutoff scores of Insomnia Severity Index-2.

## Abbreviations

AUC: area under the curve
EMA: ecological momentary assessment
ISI: Insomnia Severity Index
ISI-2: Insomnia Severity Index-2
K-CESD-R: Korean version of the Center for Epidemiologic Studies Depression Scale–Revised
PHQ-2: Patient Health Questionnaire-2
PHQ-9: Patient Health Questionnaire-9
ROC: receiver operating characteristic
YJ: Youden J

## Data Availability

The data collected and used in this meta-analysis can be requested from the corresponding author.
