# Supplementary material for: Evaluating the Modified Patient Health Questionnaire-2 and Insomnia Severity Index-2 for Daily Digital Screening of Depression and Insomnia: Validation Study
Source: JMIR Ment Health. 2023 May 22;10:e45543. doi: 10.2196/45543 (PMC10242457; doi:10.2196/45543)
Supplement: Multimedia Appendix 1 [file mental_v10i1e45543_app1.docx]

Multimedia Appendix 1. Participants with other diagnosis

|  | Males | | Females | |
| --- | --- | --- | --- | --- |
|  | N | % | N | % |
| Anxiety disorders | 9 | 27.3 | 24 | 72.7 |
| Attention deficit-hyperactivity disorder | 1 | 100 | - | - |
| Bipolar disorders | 3 | 30 | 7 | 70 |
| Bulimia nervosa | - | - | 1 | 100 |
| Insomnia disorder | 3 | 37.5 | 5 | 62.5 |
| Obsessive-compulsive disorder | 4 | 66.7 | 2 | 33.3 |
| Psychotic disorders | 2 | 66.7 | 1 | 33.3 |
| Trauma and stress related disorders | 11 | 57.9 | 8 | 42.1 |
| Total | 33 |  | 48 |  |
