# Supplementary material for: Evaluating the Modified Patient Health Questionnaire-2 and Insomnia Severity Index-2 for Daily Digital Screening of Depression and Insomnia: Validation Study
Source: JMIR Ment Health. 2023 May 22;10:e45543. doi: 10.2196/45543 (PMC10242457; doi:10.2196/45543)
Supplement: Multimedia Appendix 2 [file mental_v10i1e45543_app2.docx]

Multimedia Appendix 2. Cut-off scores of ISI-2

|  | Cut-off value | Sensitivity | Specificity | YJ |
| --- | --- | --- | --- | --- |
| Week 2 | 3.37 | 0.96 | 0.64 | 0.61 |
|  | 3.29 | 0.96 | 0.63 | 0.59 |
|  | 3.21 | 0.96 | 0.61 | 0.57 |
|  | 3.43 | 0.89 | 0.65 | 0.54 |
|  | 3.50 | 0.85 | 0.69 | 0.54 |
| Week 4 | 3.50 | 0.95 | 0.71 | 0.66 |
|  | 3.57 | 0.92 | 0.74 | 0.66 |
|  | 3.93 | 0.85 | 0.81 | 0.65 |
|  | 3.64 | 0.90 | 0.75 | 0.65 |
|  | 3.87 | 0.85 | 0.80 | 0.64 |

* YJ – Youden’s J statistic value; ISI-2 – Insomnia Severity Index-2
